# Supplementary material for: Diversity of HLA-A2-Restricted and Immunodominant Epitope Repertoire of Human T-Lymphotropic Virus Type 1 (HTLV-1) Tax Protein: Novel Insights among N-Terminal, Central and C-Terminal Regions
Source: Biomolecules. 2023 Mar 16;13(3):545. doi: 10.3390/biom13030545 (PMC10046496; doi:10.3390/biom13030545)
Supplement: Supplementary file 1 [file biomolecules-13-00545-s001.zip › biomolecules-2132802-supplementary.pdf]

Supplementary Figures

Study flowchart of Tax peptide selection and analysis

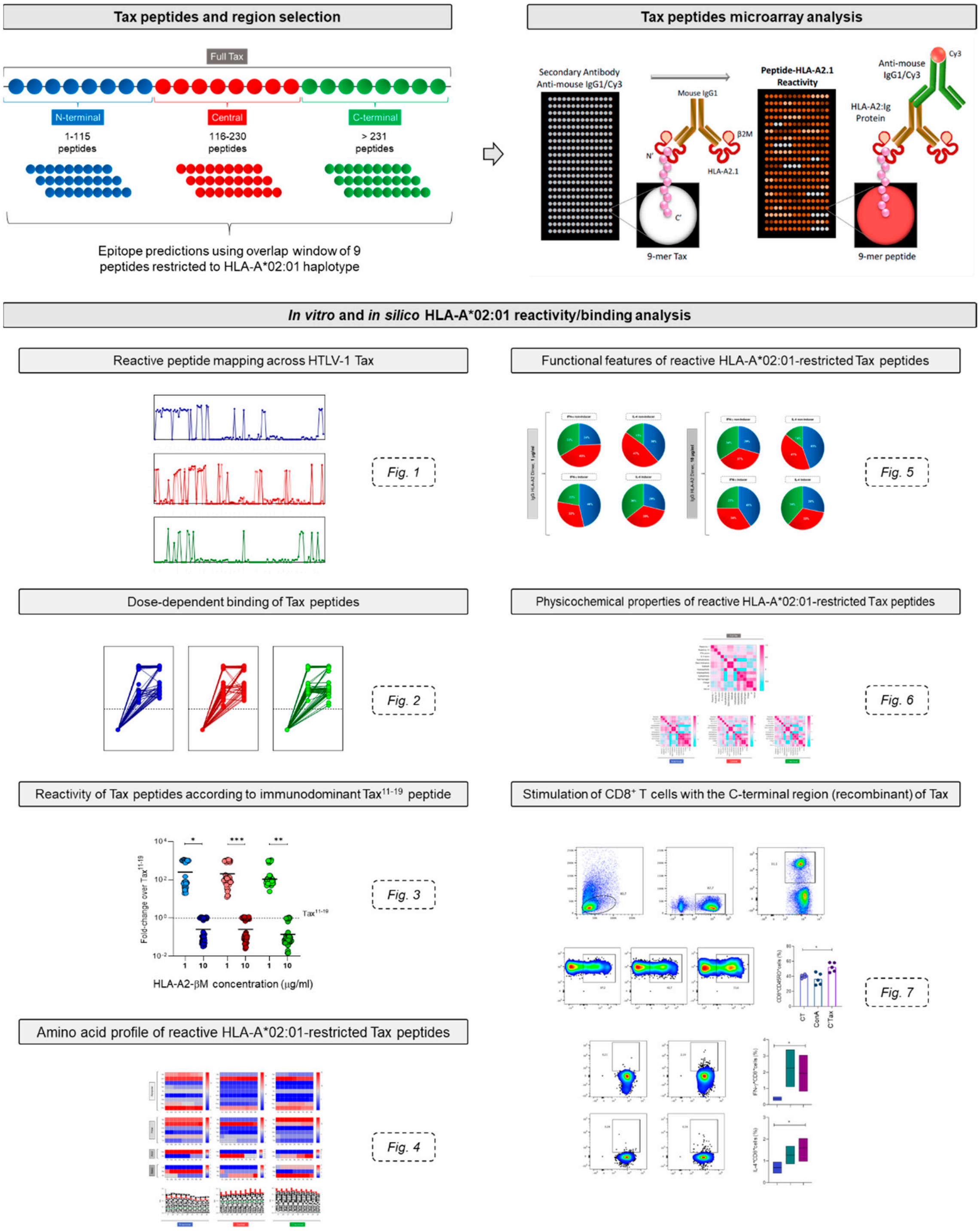

Figure S1. Flowchart representation illustrating the work outline. Strategies adopted to perform *in silico* predictions and *in vitro* assays of HTLV-1 Tax-reactive peptides. Peptide sequences were analyzed for their potential HLA-A*02:01 haplotype reactivity. Considering the immunomodulatory effect of the C-terminal portion of Tax evidenced by

in silico data, *Escherichia coli* BL21(DE3) bacteria were transformed for expression of the recombinant C-terminal region (C'Tax) of the Tax viral protein.

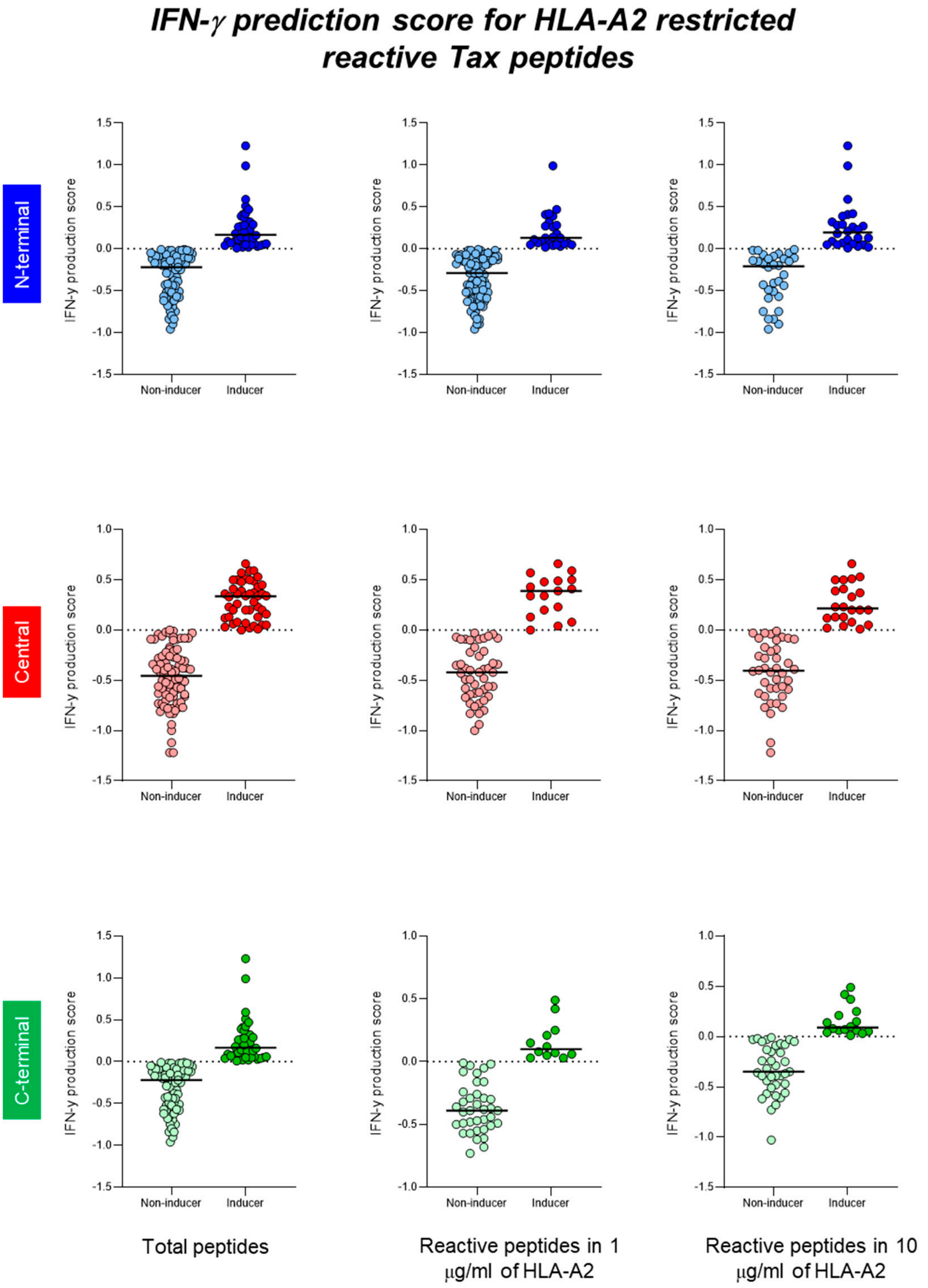

Figure S2. Predictive score of IFN-γ inducers and non-inducers of total and HLA-A2-restricted peptides at concentrations of 1 μg/mL and 10 μg/mL located in the N-terminal, Central and C-terminal regions of the Tax viral protein.

IL-4 prediction score for HLA-A2 restricted reactive Tax peptides

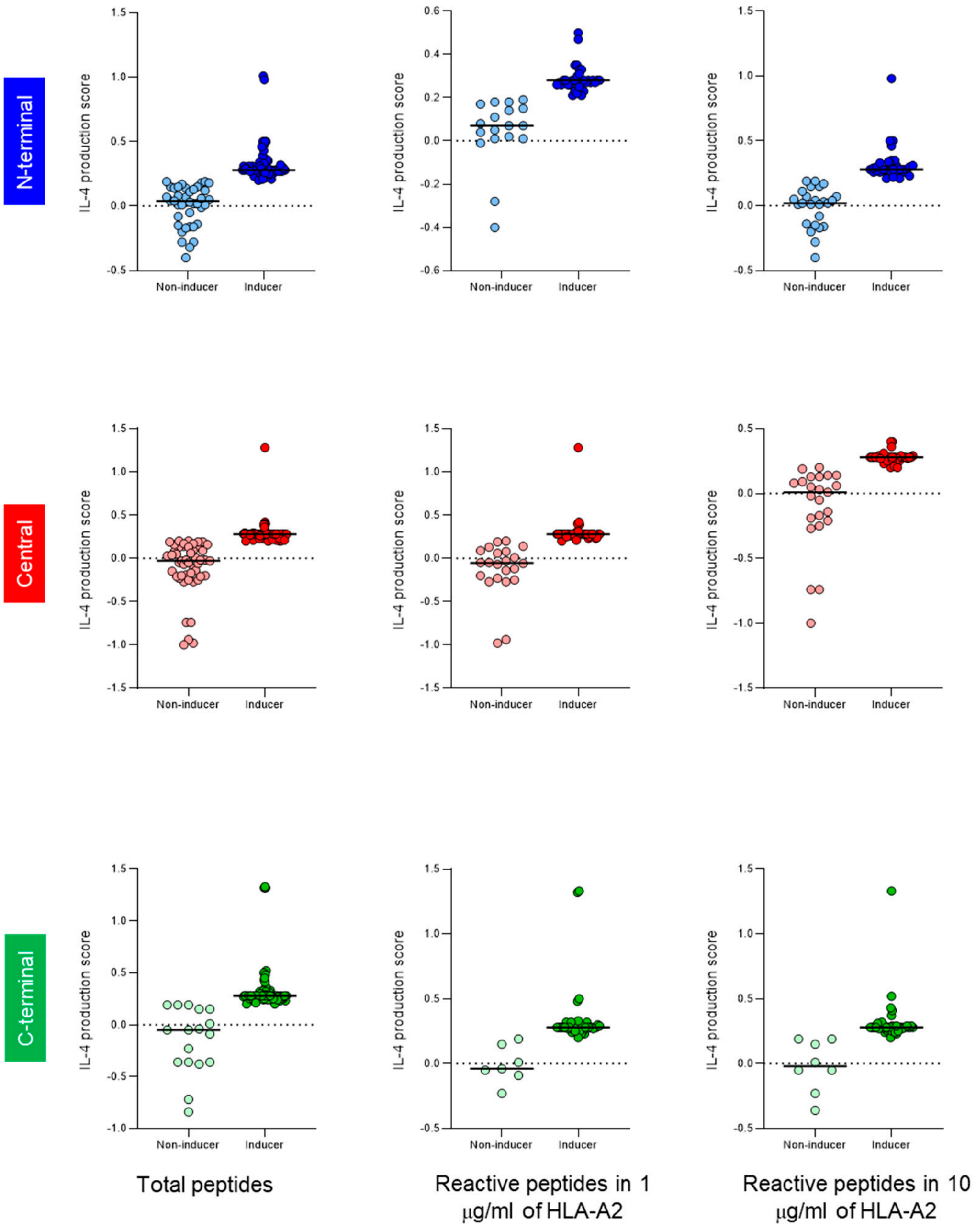

Figure S3. Predictive score of IL-4 inducers and non-inducers of total and HLA-A2-restricted peptides at concentrations of 1 µg/mL and 10 µg/mL located in the N-terminal, Central and C-terminal regions of the Tax viral protein.

Expression and evaluation of the toxicity of the C-terminal portion of the Tax protein (C'Tax recombinant protein) of HTLV-1 in human peripheral blood mononuclear cells

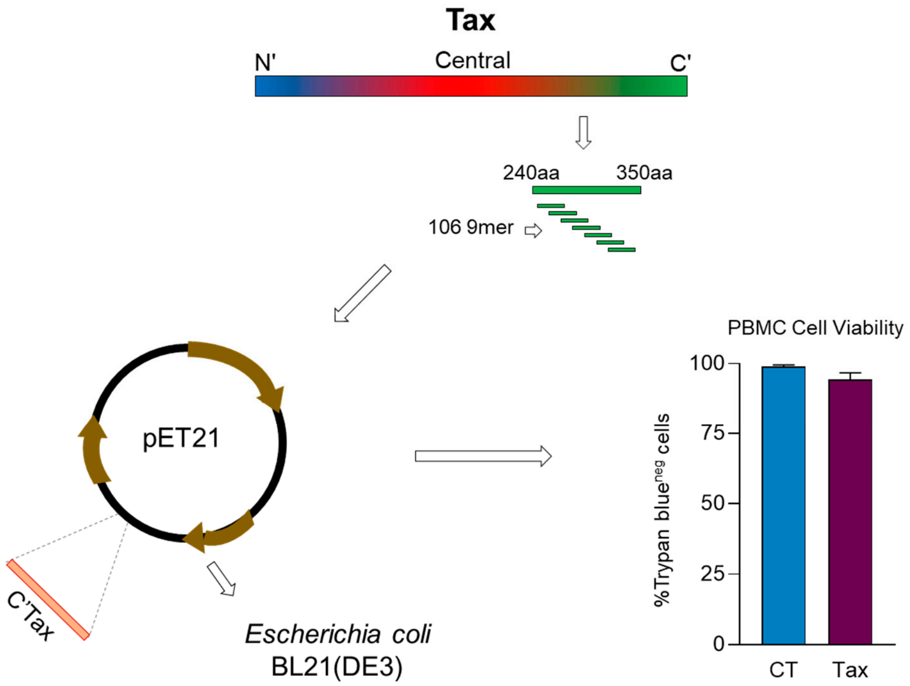

Figure S4. Production of a recombinant analogue to the C-terminal region of Tax (C'Tax). Obtaining the amino acid sequence contained between residues 240 and 350 of the polypeptide chain comprising the C-terminal portion of the HTLV-1 Tax viral protein for insertion into the pET21a vector. *Escherichia coli* BL21(DE3) bacteria are transformed with the plasmid encoding the C-terminal fragment of Tax for heterologous protein synthesis. After purification, the C'Tax recombinant was evaluated for its cytotoxic potential when maintained for 72 hours in culture with Peripheral Blood Mononuclear Cells (PBMCs) at a concentration of 2 µg/mL.
